# P075 Not all myoedema is myositis: three cases of giant cell arteritis with myoedema on MRI

**DOI:** 10.1093/rap/rkag113.105

**Published:** 2026-11-03

**Authors:** Zoe Syrimi, Jaspreet Aulakh, Devesh Mewar, Haseeb Chaudhary, Caroline Cotton

**Affiliations:** Department of Rheumatology, NHS University Hospitals of Liverpool Group, Liverpool, United Kingdom; Department of Rheumatology, NHS University Hospitals of Liverpool Group, Liverpool, United Kingdom; Department of Rheumatology, NHS University Hospitals of Liverpool Group, Liverpool, United Kingdom; Department of Radiology, NHS University Hospitals of Liverpool Group, Liverpool, United Kingdom; Department of Rheumatology, NHS University Hospitals of Liverpool Group, Liverpool, United Kingdom

## Abstract

**Introduction:**

We report three patients diagnosed with giant cell arteritis (GCA) who presented atypically with severe proximal lower-limb myalgia, constitutional symptoms, and markedly elevated inflammatory markers. magnetic resonance imaging (MRI) thighs demonstrated skeletal muscle oedema, initially raising suspicion of myositis. In all cases, however, creatine kinase was normal, muscle strength was preserved, and systemic inflammatory features predominated. This led us to consider alternative diagnoses. Temporal artery ultrasound (TAUS) was highly suggestive of GCA in each case. These cases illustrate that MRI muscle oedema is a non-specific finding and highlight GCA as a myositis mimic.

**Case description:**

A 64-year-old man presented with one week of severe thigh and flank pain, which spread to his chest, arms and head, fevers, constitutional symptoms, and bilateral leg oedema. Inflammatory markers were markedly elevated (peak CRP 478mg/L, ESR 124mm/hr). CK was normal. CT-TAP showed inflammatory changes involving the chest, lateral abdominal walls and thigh musculature. MRI thighs showed multifocal intramuscular oedema. He was treated with antibiotics until infection was excluded. Prednisolone 40mg daily resulted in transient clinical improvement before recurrence of pyrexia. A muscle biopsy was within normal limits. The patient was transferred to our hospital for PET-CT. TAUS was highly suggestive of GCA. He reported having had bilateral jaw pain, occipital pain and tenderness. He developed transient visual disturbance, so he was treated with three intravenous methylprednisolone 1g pulses followed by prednisolone 60mg daily, resulting in rapid improvement. Ophthalmology examination was normal. He has since started tocilizumab for a suspected relapse.

A 61-year-old man presented with a three-week history of bilateral proximal leg pain, fevers and rigours. Inflammatory markers were elevated (peak CRP 243mg/L, ESR 86mm/hr). CK was normal. Treatment in primary care with a course of antibiotics and prednisolone 30mg daily gave a partial response. MRI thighs showed bilateral patchy myoedema. He was referred to our myositis clinic. He had developed a headache, jaw discomfort, night sweats, and weight loss. TAUS was highly suggestive of GCA. Prednisolone 40mg daily resulted in rapid improvement.

A 59-year-old man presented with sudden-onset severe thigh pain, fevers and night sweats. Inflammatory markers were elevated (peak CRP 339mg/L, ESR 110mm/hr). CK was normal. MRI thighs showed symmetrical myoedema. He self-discharged while awaiting investigations for myositis and re-presented to our hospital. Thickened temporal arteries were noted, but he denied cranial GCA symptoms. TAUS was highly suggestive of GCA. Prednisolone 40mg daily resulted in rapid improvement.

**Discussion:**

Skeletal muscle oedema on MRI, characterised by increased T2-weighted or STIR signal, is a non-specific imaging finding with a broad differential diagnosis. Although it is often associated with idiopathic inflammatory myopathies or “myositis”, it may also be seen in infection, drug-induced or toxic myopathies, metabolic myopathies, muscular dystrophies, denervation, rhabdomyolysis, muscle infarction, trauma and malignancy. MRI findings should therefore always be interpreted within the clinical context. In our cases, MRI appearances initially misdirected investigation towards myositis. While a minority of patients with idiopathic inflammatory myopathies may have a normal CK, it should increase suspicion of a myositis mimic and prompt consideration of alternative diagnoses.

In our cases, proximal lower-limb pain and systemic inflammatory features were predominant, and classical cranial manifestations of GCA were either less pronounced, developed later or absent. TAUS is a non-invasive investigation that can establish the diagnosis of GCA quickly, avoiding unnecessary and invasive investigations.

Although polymyalgia rheumatica (PMR) commonly coexists with GCA, muscle oedema on MRI is not typical for PMR. Typical features of PMR on MRI include subdeltoid bursitis, biceps tenosynovitis, glenohumeral synovitis, hip synovitis, and trochanteric bursitis.

Reports of MRI muscle oedema in GCA are extremely sparse, with only a few cases in the literature. Our case series therefore broadens the recognised spectrum of musculoskeletal manifestations of GCA.

The mechanism underlying muscle oedema in GCA is unknown. Possible explanations include oedema due to ischaemia from vasculitis or a cytokine-mediated inflammatory response. A normal muscle biopsy from one patient suggests that the muscle oedema on MRI may occur without true muscle inflammation, although the exact origin of the MRI abnormality remains unclear.

**Key learning points:**

• Muscle oedema on MRI is a non-specific finding with a broad differential diagnosis.

• Normal creatine kinase and normal muscle strength should prompt consideration of a myositis mimic in patients with myoedema on MRI.

• Giant cell arteritis can present atypically with severe proximal lower-limb myalgia and systemic inflammatory features before classical cranial symptoms become apparent.

• Temporal artery ultrasound can rapidly establish the diagnosis of GCA in patients with unexplained systemic inflammation.

• Early recognition of atypical GCA is important to avoid unnecessary investigations and facilitate prompt corticosteroid treatment.

